# Supplementary material for: Coherent X-ray measurement of step-flow propagation during growth on polycrystalline thin film surfaces
Source: Nat Commun. 2019 Jun 14;10:2638. doi: 10.1038/s41467-019-10629-8 (PMC6570654; doi:10.1038/s41467-019-10629-8)
Supplement: Supplementary file 1 — Supplementary Information [file 41467_2019_10629_MOESM1_ESM.pdf]

Supplementary Information for “Coherent X-ray measurement of step-flow propagation during growth on polycrystalline thin film surfaces”

Headrick et al.

Supplementary Note 1

The wave vector transfer $\boldsymbol{Q}$ is resolved into a component Q_z perpendicular to the sample surface and a component $Q_{||}$ in the plane of the surface. The reflected beam appears at $Q_z = \frac{4\pi}{\lambda}\sin(\alpha_i)$ = 0.0685 Å^{-1} with α_i = 0.4° and λ = 1.28 Å. For a surface with the electron density of bulk C_{60}, a Yoneda wing is expected to appear at a critical angle α_c = 0.156°, which corresponds to $Q_z = \frac{2\pi}{\lambda}[\sin(\alpha_i) + \sin(\alpha_c)]$ = 0.0484 Å^{-1}. The specular reflection and the Yoneda wing are both visible in the scattering pattern collected during growth at 144°C shown in Supplementary Figure 1(a).

Streaks develop during the film growth at a value of $Q_{||}$ related to the correlation length $\xi(t)$ during the early stages through $Q_{corr} = 2\pi/\xi(t)$. $\xi(t)$ stops changing when the correlation length approaches the mound size. In Supplementary Figure 1(a), the vertical streak corresponds to $Q_{||} \approx Q_y = 0.00199$ Å^{-1}. From this value, we deduce a mound size of $\xi \approx 3160$ Å (0.32 μm) for growth at 144°C. The third component of the scattering vector, Q_x, is in the direction of the projection of the incident X-ray beam onto the surface. The maximum value of Q_x, which occurs at Q_z = 0.040 and Q_y = 0 is $Q_x = 1.1 \times 10^{-4}$ Å^{-1}, so that the minimum length scale probed along the x-direction is $\approx$ 55,000 Å (5.5 μm).

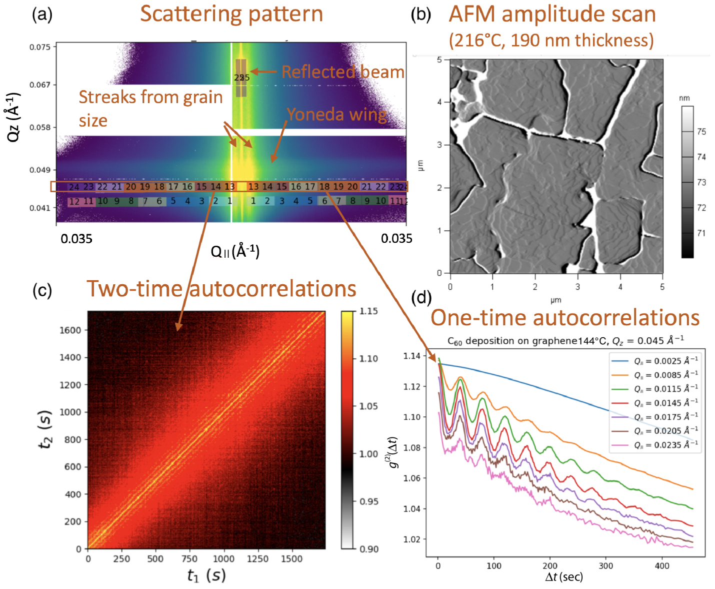

Supplementary Figure 1: Examples of the data and analysis. **(a)** Grazing Angle Small Angle X-ray Scattering (GISAXS) pattern. Regions of interest (ROIs) 13 through 23 are the areas on the image that were used for the coherent analysis presented in the manuscript. **(b)** Atomic Force Microscopy amplitude image of one of the samples after the deposition, showing mounds with molecular-height terraces. **(c)** Two-time correlations for a series of 9000 images acquired at 2.5 frames per second and subsequently binned to 1.6 sec per frame. Only a narrow range of times during the time when the average scattering pattern was not changing with time is shown in the image. **(d)** One-time correlations for the steady-state regime is shown for a range of $Q_{||}$ and a single Q_z.

The entire pattern, including the streaks and the Yoneda wing contain speckle due to the coherence of the X-rays. The speckle changes in time during the film growth as the surface evolves. Static analysis of $I(Q,t)$ is carried out by averaging over regions that are larger than the size of individual speckles, so that the speckles are effectively averaged out. This analysis is carried out below the Q_z where the Yoneda wing intensity is maximum to improve surface sensitivity.

Dynamic analysis of the data is carried out at Q_z values below the Yoneda wing in order to maximize surface sensitivity. Surface sensitivity is important since it suppresses oscillatory correlations related to the film growth velocity. This effect is related to mixing of the surface scattering with bulk scattering,[1] and it becomes particularly noticeable for films grown at lower temperatures that tend to have more defects and a correspondingly larger bulk scattering signal.

Analysis tools for X-ray Photon Correlation Spectroscopy (XPCS) developed at NSLS-II for the Coherent Hard X-ray beamline (CHX, 11-ID) were used to calculate the correlations presented in the manuscript.[2] Since the intensity changes with time in the early stages of the deposition, the standard method of calculating the average signal for normalization of the correlations by averaging over time becomes inaccurate. We have employed a

different procedure: In order to produce the two-time plot in Supplementary Figure 1(c), a Savitsky-Golay filtering[3] is performed to produce a smoothened average intensity profile $\widetilde{I(\boldsymbol{Q})} = \widetilde{\langle I(\boldsymbol{Q},t)\rangle_t}$ that is comparable to that which would be measured under low-coherence conditions where the speckles are not resolved. In this expression, $\widetilde{\langle\cdots\rangle_t}$ denotes a time-average followed by Savitsky-Golay smoothening with a window size of 11, which can be compared to the speckle size of 2-3 pixels. The normalization of the two-time correlation function is then performed using $\langle I'(\boldsymbol{Q},t)\rangle_Q = \langle I(\boldsymbol{Q},t)/\widetilde{I(\boldsymbol{Q})}\rangle_Q$ instead of a simple time-averaged intensity.

One-time correlation functions $g^{(2)}(\Delta t)$ are calculated by two methods: The first method is to average the two-time correlations over times where the intensity is at steady-state, and the second method is to use Equation 2 in the main text using the time-averaged $\langle I(t)\rangle_t$ for the normalization. We confirmed that both methods produce comparable results as long as $I(t)$ is unchanging for the time interval included in the analysis. Results computed by the second method are shown in Supplementary Figure 1(d), Supplementary Figure 2, Supplementary Figure 5, and in Figure 3 of the main text.

Supplementary Note 2

Supplementary Figure 2 shows examples of oscillatory correlations corresponding to step flow growth at several substrate temperatures. It is observed that the oscillations have a maximum amplitude at a particular $Q_{||}$, which we refer to as $Q_{\max}$. The length scale $2\pi/Q_{\max}$ is interpreted as being approximately equal to the mean terrace length on the tops of the mounds. Note that the speckle contrast is somewhat reduced in Supplementary Figure 2(a),(d),(e) due to vibrations from the vacuum pumping system. The speckle contrast is reduced at high $Q_{||}$ in Supplementary Figure 2(c) due to shot noise as a results of poor counting statistics.

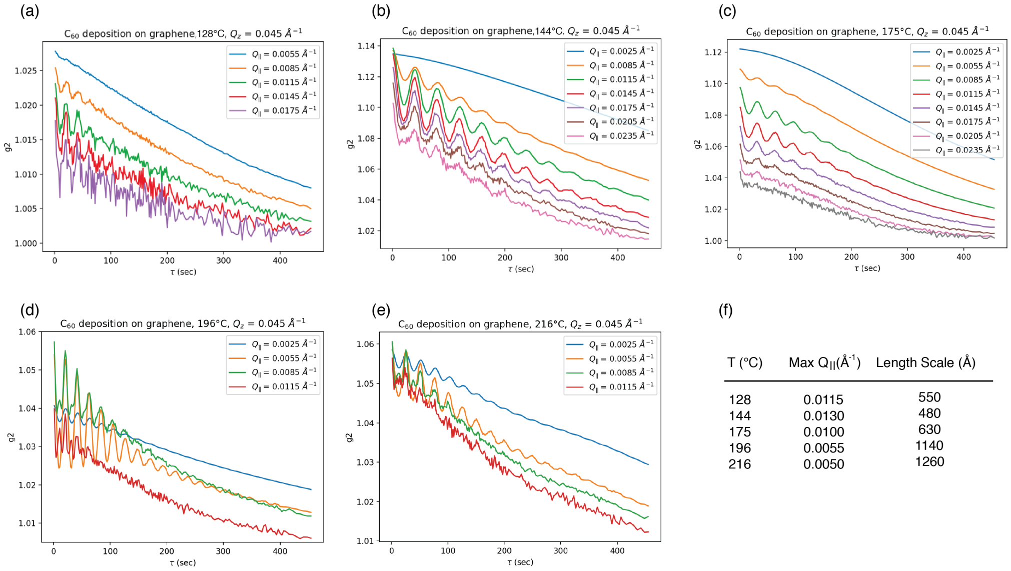

T (°C)	Max $Q_{||}$(Å^{-1})	Length Scale (Å)
128	0.0115	550
144	0.0130	480
175	0.0100	630
196	0.0055	1140
216	0.0050	1260

Supplementary Figure 2: $g^{(2)}(\Delta t)$ curves for thin films deposited at different temperatures. **(a)** 128 **(b)** 144, **(c)** 175, **(d)** 196, and **(e)** 216°C. **(f)** table summarizing the $Q_{||}$ values where the oscillation amplitude is the largest for each growth temperature.

Supplementary Figure 3 shows a plot of the terrace length computed from $2\pi/Q_{max}$ versus the inverse of the growth temperature. Fitting results to an Arrhenius form yield an energy of 0.21 eV, as shown in the figure. The terrace length is mainly controlled by the critical radius for nucleation of a new layer. Nucleation occurs when the radius of the top terrace of the mound has reached R_c. The critical radius itself can be related to the effective rate of interlayer transport $\nu' = \nu_0'\exp(-E_S/k_BT)$ through $R_c \propto (\nu')^{1/5}$.[4] This relation implies that the Ehrlich-Schwoebel barrier E_s can be obtained from the temperature dependence of Q_{max}. The procedure yields the value E_S = 1.05±0.55 eV, since the slope obtained in Supplementary Figure 3 is only 1/5 of E_S. Mound formation should occur when E_S is larger than the barrier for surface diffusion E_d. We can also compare our experimental value to the value of $E_S = 0.65$ eV obtained in an earlier study through Kinetic Monte-Carlo simulations of layer-by-layer oscillations.[5]

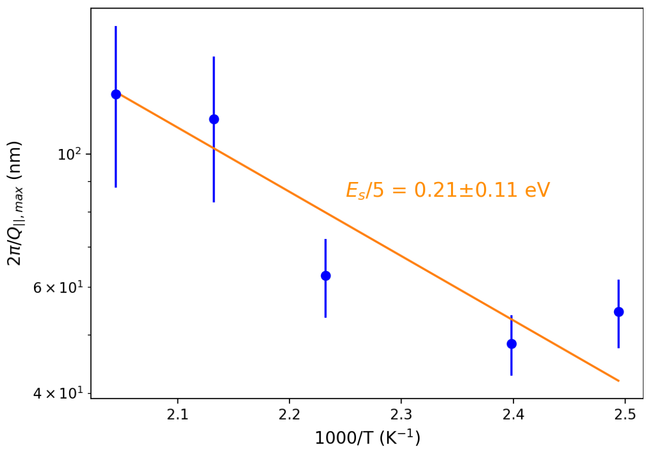

Supplementary Figure 3: Arrhenius plot of the terrace length derived from autocorrelations during growth.

Supplementary Note 3

The height image derived from Atomic Force Microscopy data is a direct measurement of the surface topography. Supplementary Figure 4(a) shows that the mounds are bounded by deep grooves. Supplementary Figure 4(b) shows the amplitude image of the same scan area. It highlights certain details of the surface topography such as step-edges. These are the complete images corresponding to Figure 4(a) in the main text.

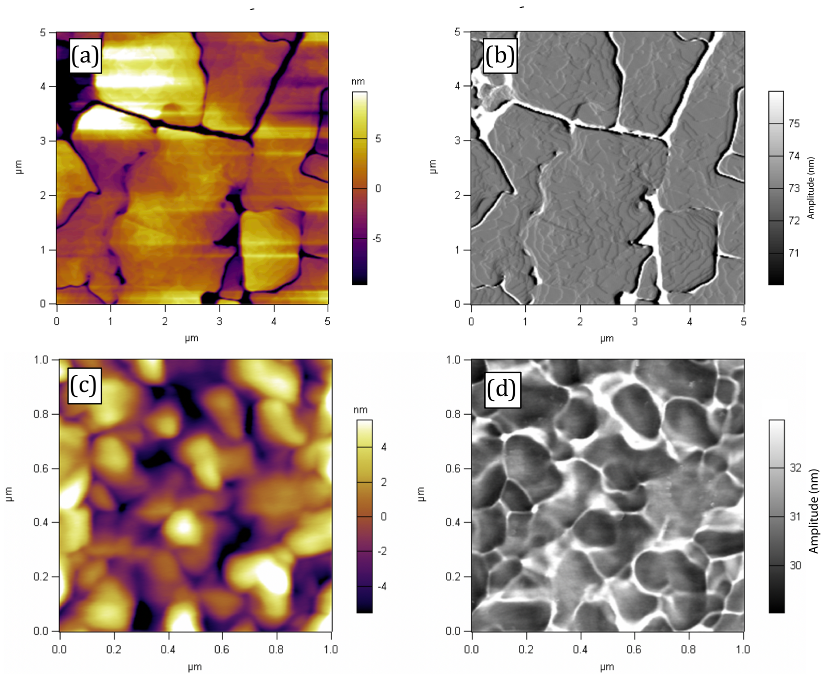

Supplementary Figure 4: Comparison of AFM imaging modes for the same scan. **(a)** height image for growth at 216°C. **(c)** height image for growth at 106°C. **(b,d)** cantilever amplitude images corresponding to **(a)** and **(c)** respectively.

Supplementary Note 4

Supplementary Figure 5 shows the steady-state autocorrelation results from Figure 3(a) of the main text with fitting results using the model of Equations (3) and (4) of the main text. The table shows the fitting parameters for each curve. The parameter I_2 does not appear in the table since it set to zero for each curve shown.

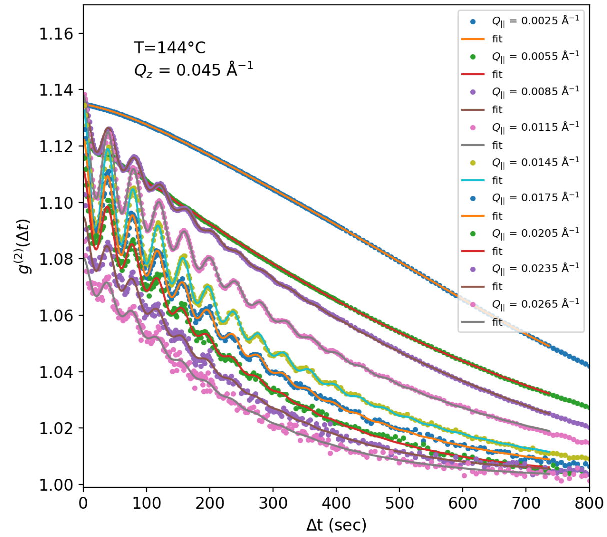

	$Q_{\|\|}$	*baseline*	I_0	Γ_0	I_1	Γ_1	ω_1	n
**0**	0.0025	0.980000	0.392047	0.000729	0.001324	0.009427	0.020000	1.487810
**1**	0.0055	0.989827	0.362575	0.000833	0.002291	0.014530	0.144403	1.165902
**2**	0.0085	0.980000	0.386201	0.000826	0.011281	0.009457	0.151968	1.035946
**3**	0.0115	1.002201	0.343493	0.001328	0.025974	0.007049	0.155781	1.114922
**4**	0.0145	1.002653	0.330503	0.001648	0.031557	0.006315	0.157800	1.151454
**5**	0.0175	1.003468	0.318971	0.001871	0.026878	0.007194	0.158558	1.190456
**6**	0.0205	1.002574	0.308679	0.002053	0.021445	0.008517	0.159028	1.204065
**7**	0.0235	1.003846	0.286976	0.002287	0.015235	0.008739	0.159063	1.240448
**8**	0.0265	1.002422	0.268905	0.002364	0.010678	0.008564	0.159102	1.232009

Supplementary Figure 5: Plot of the autocorrelations for C_{60} growth at 144°C with fitting results and fitting parameters.

Supplementary Note 5

Supplementary Figure 6 shows intensity data for a deposition at 144°C, the same data used to make the correlation plots shown in Figure 2 and Figure 3(a) of the main text. The growth shutter was opened at 40 sec and stopped at 3200 sec. The calibrated deposition rate was 11.1 Å/min. The period of the Kiessig oscillations in the reflected beam calculated from $T = 2\pi/(v_{growth}Q_z)$ is 496 sec. This period is in good agreement (within 3%) with the period of oscillations in Supplementary Figure 6, (481 sec.). Note that this period is more than an order of magnitude larger than the monolayer deposition time: Assuming a layer spacing of 8 Å, the monolayer formation time is 43 sec. The intensity of the diffuse scattering is also shown in Supplementary Figure 6. It does not exhibit oscillations. Instead, there is a broad peak in the early stages of growth. This peak reaches a maximum at about 450 sec, corresponding roughly to the time that the step-flow oscillations first appear in Figure 2 of the main text.

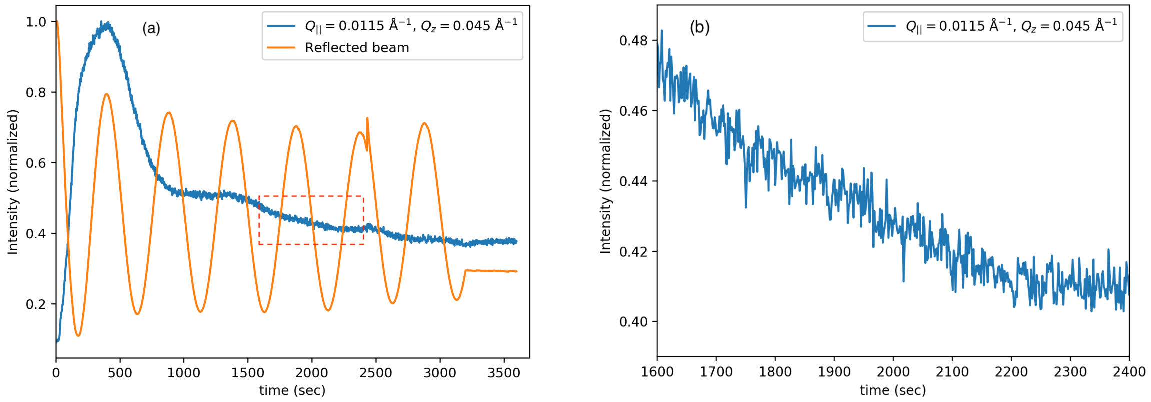

Supplementary Figure 6: Plot of the reflected beam intensity and diffuse intensity as a function of time for C_{60} growth at 144°C. **(a)** Full scan, and **(b)** zoomed to the red dashed area in **(a)**.

Supplementary Note 6

Scaling analysis for room temperature deposition using the correlation length $\xi(t) = 2\pi/ Q_{corr}$ is shown in Supplementary Figure 7. An exponent of 1/z = 0.45 is obtained in the early time. However, $\xi(t)$ stops changing at later times. This behavior is interpreted as the correlation length being limited by the size of mounds. We also note that the value of the exponent γ extracted in Fig 7(a) is -3.70. An interpretation of this value is that it is characteristic of the asymptote of the scattering intensity of nearly hemispherical mounds, in which case $\gamma \approx -4$ is expected. Scattering from mounds, as discussed in the main text is therefore considered to be a reasonable interpretation of the data.

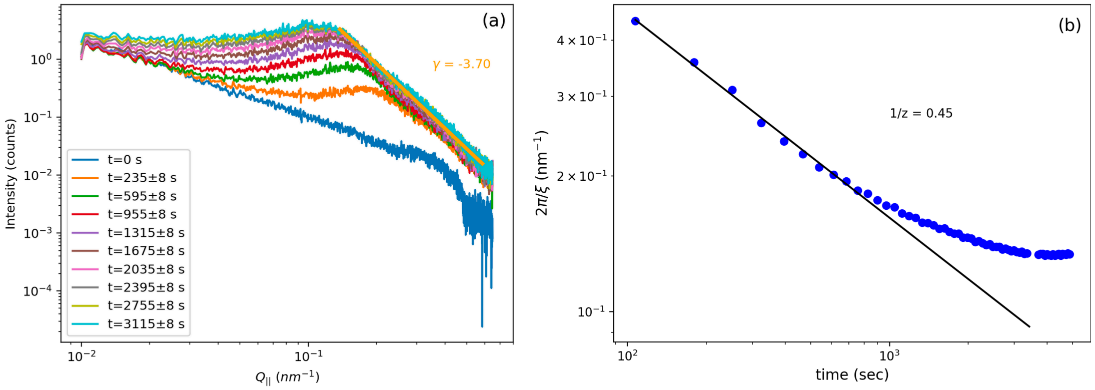

Supplementary Figure 7: Static analysis for a C_{60} thin film deposited at room temperature. **(a)** Intensity $I(t)$ vs. $Q_{||}$ at different growth times. Q_z is 0.045 Å^{-1}. **(b)** Q_{corr} vs. time extracted from $I(t)$.

Supplementary Note 7

Structure factors for the Zeno growth model were calculated by considering the mound to be a stack of monolayer-height disks with radius R_j, where j is counted from the bottom of the mound to the top. The structure factor for the stack is calculated from:

$$F_j(\mathbf{Q}, t) = \frac{2J_1(Q_{||}, R_j(t))}{Q_{||}R_j(t)} e^{iQ_z z_j} \quad (1a)$$

$$I(\mathbf{Q}, t) = \left| \sum_{j=1}^{M(t)} F_j(\mathbf{Q}, t) \right|^2 \quad (1b)$$

where $J_1(x)$ is the Bessel function of the first kind and $M(t)$ is the total number of layers in the mound at time t. The phase factor $\exp(iQ_z z_j)$ is applied to take into account the height of each layer in the direction perpendicular to the surface. Supplementary Equation (1a) is also multiplied by an exponential damping factor $\exp[\alpha(z_j - z_0)]$, only for the lower layers $z_j \leq z_0$, where z_0 is the height of the highest layer with R = 200. This factor was applied in order to simulate the absorption of X-rays as they penetrate beneath the surface in grazing incidence. Correlations were derived from the simulated intensity profiles during growth of the last 10 layers using Equations (1) and (2) in the main text. Examples of the intensity profiles during the simulated growth of the mound are shown in Supplementary Figure 8. These profiles exhibit a fast modulation that corresponds to the overall lateral extent of the mound, and this modulation is convolved with weak broader peaks that correspond to the mean step spacing. The fine features correspond to the speckle in this one-dimensional model. Their intensity varies with both $Q_{||}$ and time. One of the broad peaks is indicated by a red dashed box in Supplementary Figure 8(a), and an expanded view of it is shown in Supplementary Figure 8(b). Correlations shown in Figure 6 of the main text were generated from regions of the $I(\boldsymbol{Q}, t)$ profiles centered at 0.39 inverse lattice units [Figure 6(a,c)] and at 0.91 inverse lattice units [Figure 6(b,d)]. We note that the weak peaks corresponding to the step spacing were not observed in the experimental data since the steps are more disordered than in the model, and there is a range of mound sizes rather than the single mound simulated in the model.

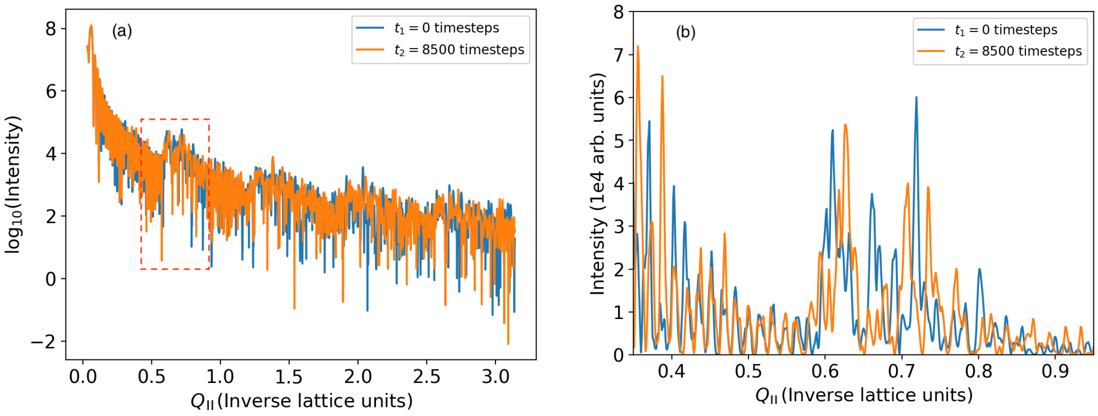

Supplementary Figure 8: Calculated intensity for the local step flow model (Zeno model) described in the main text. **(a)** Intensity at two different times during the simulated growth. **(b)** Comparison of two different times for the narrower range of $Q_{||}$ values indicated by the dashed box in **(a)**.

Supplementary References

1 Ulbrandt, J. G., Rainville, M. G., Wagenbach, C., Narayanan, S., Sandy, A. R., Zhou, H., Ludwig Jr, K. F. & Headrick, R. L. Direct measurement of the propagation velocity of defects using coherent X-rays. *Nature Physics* **12**, 794, doi:https://doi.org/10.1038/nphys3708 (2016).

2 Zhang, Y., Lhermitte, J., Rakitin, M., Allan, D. & Caswell, T. A. in *NSLS-II-CHX/pyCHX: Release for archiving and DOI-issuing, archive of NSLS-II-CHX github repository,* https://doi.org/10.5281/zenodo.1313850 (zenodo.org, July 17, 2018).

3 Savitzky, A. & Golay, M. J. Smoothing and differentiation of data by simplified least squares procedures. *Analytical chemistry* **36**, 1627-1639, doi: https://doi.org/10.1021/ac60214a047 (1964).

4 Krug, J. & Kuhn, P. in *Atomistic Aspects of Epitaxial Growth* (eds Miroslav Kotrla, Nicolas I. Papanicolaou, Dimitri D. Vvedensky, & Luc T. Wille) 145-163 (Springer Netherlands, 2002).

5 Bommel, S., Kleppmann, N., Weber, C., Spranger, H., Schäfer, P., Novak, J., Roth, S., Schreiber, F., Klapp, S. & Kowarik, S. Unravelling the multilayer growth of the fullerene C_{60} in real time. *Nature communications* **5**, 5388, doi: https://doi.org/10.1038/ncomm6388 (2014).
